# Novel Nonablative Radiofrequency Approach for the Treatment of Anal Incontinence: A Phase 1 Clinical Trial

**DOI:** 10.7759/cureus.40500

**Published:** 2023-06-16

**Authors:** Patrícia Lordêlo, Juliana Barros, Claudia Liony, Cristiane Maria Carvalho C Dias, Janine Ferreira, Priscila G Januário, Luana N Matos, Camila O Muniz, Laizza S Silva, Cristina Brasil

**Affiliations:** 1 Obstetrics and Gynecology, Pelvic Floor Care Center, Bahiana School of Medicine and Public Health, Salvador, BRA; 2 Physical Medicine and Rehabilitation, Pelvic Floor Care Center, Bahiana School of Medicine and Public Health, Salvador, BRA; 3 Physical Medicine and Rehabilitation, Bahiana School of Medicine and Public Health, Salvador, BRA; 4 Physiotherapy, Pelvic Floor Care Center, Bahia State University, Salvador, BRA; 5 Medicine, Pelvic Floor Care Center, Bahiana School of Medicine and Public Health, Salvador, BRA; 6 Medicine, Pelvic Floor Care Center, Salvador University, Salvador, BRA; 7 Physiotherapy, Pelvic Floor Care Center, Bahiana School of Medicine and Public Health, Salvador, BRA

**Keywords:** anal incontinence, nonablative radiofrequency, patient satisfaction, treatment, woman, radiofrequency, fecal incontinence

## Abstract

Objective: We aimed to describe the action, impact on quality of life, and side effects of perianal nonablative radiofrequency (RF) application in the treatment of anal incontinence (AI) in women.

Methods: This was a pilot, randomized clinical trial conducted between January and October 2016. We enrolled women who consecutively attended the Attention Center of the Pelvic Floor (CAAP) with complaints of AI for more than six months. Nonablative RF was applied to the perianal region of the participants using Spectra G2 (Tonederm®, Rio Grande do Sul, Brazil). The reduced or complete elimination of the need for protective undergarments (diapers and absorbents) was considered a partial therapeutic response.

Results: Nine participants reported treatment satisfaction, while one reported dissatisfaction with the nonablative RF treatment of AI based on the Likert scale. No patient interrupted treatment sessions because of adverse effects, although adverse effects occurred in six participants. However, the clinical and physical examination of the participants with burning sensations showed no hyperemia or mucosal lesions.

Conclusions: This study showed a promising reduction of fecal loss, participant satisfaction with treatment, and improved lifestyle, behavior, and depression symptoms with minimal adverse effects.

## Introduction

Anal incontinence (AI) is defined by the International Continence Society (ICS) as an involuntary elimination of gas or stool in the liquid or solid form [1,2]. AI shows a similar prevalence worldwide in both sexes varying from 18% to 47% [3], while in Brazil, the prevalence found is 10% in women [4]. This variation in prevalence is mainly attributable to the terminology and criteria used in the definition of AI [5].

Despite the high prevalence, less than half of the patients seek therapy because of the associated embarrassment in describing the symptoms, which invariably is accompanied by negative psychological experiences [6]. Therefore, AI causes discomfort, social isolation, and low self-esteem, which lowers the quality of life (QoL) index in these patients [7].

The available treatments are varied, and their success rates vary from 18% to 70%, depending on the severity and specific cause of the condition [8,9]. Between them, there is surgery, indicated in severe cases, and pharmacological and physiotherapeutic treatment, indicated in moderate and slight cases [10]. The physiopathology of AI is multifactorial, and it is known to be the relation of risk of perianal muscle damage [11]. Thus, one treatment option for controlling the loss of stool is based on pelvic floor rehabilitation, which consists of exercises to improve the tone and strength of the pelvic floor muscles [12].

However, pelvic floor muscle training does not benefit all patients with AI since its pathophysiology may also involve the smooth muscle of the internal sphincter, which does not improve its function with voluntary contractions [13]. Based on this observation, the use of ablative radiofrequency (RF) has emerged, which is targeted at increasing collagen production and, consequently, improving the passive continence of the anal sphincter [7,8]. However, this technique requires the use of antibiotics, analgesics, and insertion of needles in the anal canal, and is associated with adverse effects such as sphincter lesions, anal mucosa necrosis, pain, and bleeding [14].

Nonablative RF has been used on the perianal area of swine, and histological examinations of the internal anal sphincter have demonstrated the hypertrophy of smooth muscle and an increase in the ratio of collagen type I to III [15]. However, there are no human studies of nonablative RF in the treatment of AI and, currently, no data on non-invasive treatments using RF to remodel the restructure of the internal anal sphincter of human beings have been reported. Despite these shortcomings, the safety of the technique and clinical benefits were shown in the genital region in the treatment of urinary incontinence in women [1]. In that study, the treatment of urinary incontinence with nonablative RF showed a few adverse effects and reduced urinary loss.

Therefore, we hypothesized that the application of nonablative RF to the perianal site may reduce stool loss and gas with few side effects. Thus, the objective of this study was to investigate the action, impact on the QoL, and side effects of nonablative RF applied to the perianal site for the treatment of AI in women.

## Materials and methods

Study design and ethics

This study was a pilot phase 1 clinical trial, conducted between January and October 2016. The study was approved by the Ethics Committee for Research of the Escola Bahiana de Medicina e Saúde Pública (CAAE: 43462915.8.0000.554 4) and it adhered to the recommendations of the Declaration of Helsinki. The trial was registered at ClinicalTrials.gov (NTR: 03147729) and all the subjects agreed to participate in the study after signing the consent form.

Inclusion and exclusion criteria

The eligibility criterion was all women who consecutively attended the Attention Center of the Pelvic Floor (CAAP), with complaints of AI for more than six months. We excluded women who were pregnant; used intrauterine devices, pacemakers, or an implantable cardioverter defibrillator; had a diagnosis of anal fissure, fistula, anal abscess, rectal prolapse, and neurological conditions; and alterations that resulted in fecal incontinence or any of the following symptoms: pain, burning, pruritus, sensation of moisture or aeration in the anal region, and bleeding or lesion of the anal mucosa.

Subject evaluation

Patients were initially required to complete a questionnaire to provide demographic data and clinical history, including obstetric history, previous surgery, hormonal status, and presence of stool/gas loss. The subjects completed two self-administered questionnaires to analyze the stool loss (Fecal Incontinence Severity Index (FISI)) and impact on the QoL (Fecal Incontinence Quality of Life (FIQL)), and both were self-enforcing.

Description of procedure

Nonablative RF was applied to the perianal region by a single professional, using the Spectra G2 device (Tonederm®, Rio Grande do Sul, Brazil). The electromagnetic wave generator with a frequency of 0.5 MHz was connected to a handle with a 0.5 cm diameter metal electrode (active electrode) and metal plate (passive electrode). Each nude participant was placed in the left lateral decubitus position with their lower limbs flexed and the passive plate engaged in the left hip region. The active electrode was placed in contact with the perianal region, and circular, continuous movements were made, after the beginning of the wave passage. A digital infrared thermometer (ICEL TD-925, ICEL, Manaus, Brazil) was used to monitor the temperature up to 39-41°C, after which the movements were maintained for two minutes using a stopwatch coupled to the radiofrequency equipment used in the intervention [16].

The procedure lasted for five minutes at most. This protocol consists of five sessions, performed weekly, and a similar procedure was used on the urethral meatus and female external genitalia [17,18]. During the treatment, no other therapeutic procedures were performed. Furthermore, women who had continuously used other medication (such as hormones or laxatives on a regular basis) were instructed to maintain their usual dose over the study period.

Evaluation of therapeutic response

The assessments were performed seven days after the completion of treatment and then one month later. The FISI was used to evaluate the objective response based on the frequency of episodes of incontinence ranging from zero to 61 points, which depicted the scale from the lowest to highest severity of the loss [19]. FISI scores above 30 are more likely to be associated with impaired QoL than scores of 30 and below [20].

The reduction or complete elimination of the need for protective undergarments (diapers and absorbents) was considered a partial therapeutic response [21], since it suggests that there is less fecal loss, therefore, improvement in symptoms, which will result also in cost reduction.

To evaluate the impact of nonablative RF on AI, we assessed the FIQL parameters: lifestyle, behavior, depression, and embarrassment. The scores varied from one to four, where one is the worst state and four indicated the best QoL [22].

The subjective assessment was verified based on the level of patient satisfaction, using the question, "What is your level of satisfaction with nonablative RF treatment?" and the response was quantified using a five-point Likert scale, which classified the response to treatment as (1) unsatisfied, (2) not satisfied, (3) unchanged, (4) satisfied, and (5) very satisfied. To describe the adverse effects, the participants were questioned after each RF session about the following symptoms: pain, burning, pruritus, a sensation of moisture, or aeration in the anal region. The procedure was considered unsafe when bleeding and/or lesion of the anal mucosa were presented and/or when the session needed to be interrupted by any of the signs and/or symptoms described.

Statistics

The Statistical Package for the Social Sciences (SPSS) version 17.0 for Windows (SPSS Inc., Chicago, IL) was used for the descriptive and numerical analysis of the data. The normality of the variables was verified using the Kolmogorov-Smirnov test, descriptive statistics, and graphical analysis. The results are presented in tables and figures.

In the descriptive analysis, the categorical variables, including the number of pregnancies, urogynecological surgery, hormonal status, type of loss (solid, liquid, or missing), and protective garments (cloth, diaper, or absorbent), were expressed in absolute values, while the numerical variable (age) was expressed as mean ± standard deviation (SD) because it had a normal distribution.

## Results

The mean age of the participants was 51.90 ± 11.50 years, and their clinical characteristics are shown in Table 1. None of the participants used laxatives. Hormones and other drugs used are described in Table 1.

**Table 1:** Sociodemographic and clinical characteristics of 10 participants with anal incontinence (AI) subjected to nonablative radiofrequency (RF) treatment of the perianal region. Salvador, Bahia, Brazil, 2016.

ID	Age (years)	Gestations	Urogynecologic surgeries	Protection use	Hormonal status	Type of loss
01	60	7	Perineoplasty	Yes	Menopause	Liquid stools and flatus
02	55	5	No	Yes	Menopause	Liquid stools and flatus
03	62	2	Hysterectomy plus perineoplasty plus cesarean section	Yes	Menopause	Liquid and solid stools, and flatus
04	66	2	Hemorrhoidectomy plus cesarean section	No	Menopause	Solid stools
05	56	3	Cesarean section	Yes	Menopause	Liquid stools and flatus
06	41	3	Perineoplasty	Yes	Menacme	Solid stools
07	55	3	Cesarean plus perineoplasty	No	Menopause	Liquid stools and flatus
08	52	3	No	No	Menopause	Liquid stools
09	45	2	Hysterectomy plus perineoplasty plus hemorrhoidectomy	Yes	Menopause	Liquid and solid stools, and flatus
10	27	2	No	Yes	Menacme	Solid stools

**Figure 1:**
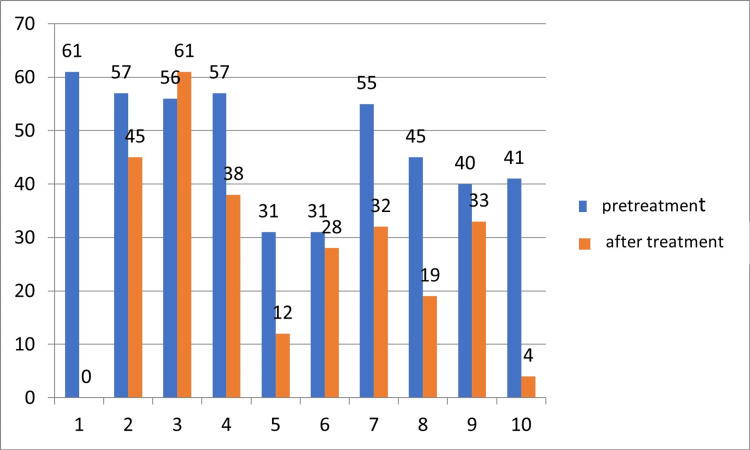
Before and after assessment by objective criteria for evaluating the response of the severity of anal incontinence of 10 participants submitted to the application of nonablative radiofrequency in the perianal region. Salvador, Bahia, Brazil, 2016. FISI: Fecal Incontinence Severity Index. FISI scores range from 0 to 61. Values ​​above 30 = AI severity.

The immediate clinical response to perianal nonablative RF in relation to the severity of AI was measured using FISI, and eight participants exhibited a decrease in the AI severity. Seven participants (participant 1, participant 4, participant 5, participant 7, participant 8, participant 9, and participant 10) had a reduction of at least four points on the FISI score. Furthermore, five participants achieved a score that indicated a low severity of AI. Participant 1 was the only one who did not return for all evaluations after treatment, so we presented her post-treatment result for the FISI score as missing data (Figure 1).

Of the seven participants who used protective undergarments (such as diapers, absorbents, and cloth), four participants stopped using them after treatment with nonablative RF. That was considered a partial therapeutic response.

The FIQL questionnaire administered after five sessions of nonablative RF revealed that six participants showed improvement of the QoL in lifestyle, behavior, and depression. In the five embarrassment parameters, the participants showed improved QoL scores, and the results are described in Table 2.

**Table 2:** Before and after evaluation of the quality of life (QoL) responses of anal incontinence (AI) of 10 participants subjected to nonablative radiofrequency (RF) treatment of the perianal region using objective criteria. Salvador, Bahia, Brazil, 2016. FIQL: Fecal Incontinence Quality of Life. FIQL: 1 = worst quality of life and 4 = best quality of life.

Identification	Fecal incontinence-related quality of life																						
	FIQL: lifestyle						FIQL: behavior					FIQL: depression					FIQL: embarrassment				FIQL: total score		
	Before treatment		After treatment			Before treatment			After treatment		Before treatment			After treatment		Before treatment		After treatment				Before treatment		After treatment
	1	3.6		3.1			4			4		3			2.7		1.3		1.3			11.9	11.1	
2	2.6		3.0			2.5			2.7		2.7			2.1		1.6		2.6			9.4	10.4	
3	1.7		3.1			1.3			3.1		1.4			2.1		1.0		2.3			5.4	10.6	
4	3.7		3.5			3.2			2.8		3.0			3.3		2.0		2.3			11.9	11.9	
5	3.8		4.0			2.6			3.4		2.8			2.8		2.6		3.6			11.8	13.8	
6	3.9		3.7			3.3			2.3		2.7			2.8		2.0		1.6			11.9	10.4	
7	3.8		4.0			3.1			3.3		2.9			3.1		3.3		2.6			13.1	13	
8	3.4		4.0			3.5			3.6		3.5			3.8		3.8		3.6			14.2	15	
9	1.8		1.0			1.6			1.6		1.3			1.0		1.0		1.0			5.7	4.6	
10	2.8		3.8			2.6			3.4		3.0			3.4		3.3		3.6			11.7	14.2	

The Likert scale revealed that nine participants reported being satisfied with the treatment while one reported dissatisfaction with the nonablative RF treatment in the perianal region.

During the performance of the technique, there were reports of adverse effects in six participants. Four participants reported a burning sensation, including one who also reported a "sandy" discomfort in the anal region and another who reported a moist anal sensation. However, the clinical or physical examination of the participants with burning sensations showed no hyperemia or mucosal lesions. No patient interrupted the session or protocol because of the adverse effects. Another adverse effect reported by two participants was pruritus. One of the participants who experienced pruritus reported a feeling of vaginal heaviness and constipation during treatment and was advised to consult their physician who recommended the use of Muvinlax® laxative.

## Discussion

The study investigated an innovative technique of nonablative RF for the treatment of AI and we obtained promising results. There was a reduction in fecal losses, as evidenced by decreased use of protection and improved FISI. We hypothesized that the clinical effect was mediated by the induction of collagen production by controlled heating at a temperature ranging from 39ºC to 41ºC. The heating causes an acute inflammatory process with activation of the fibroblasts and a consequent increase in the production of collagen and elastin [23,24]. This phenomenon may induce morphological alterations of the sphincteric muscular structure, which contributes to the mechanism of AI.

In an experimental study, the anal sphincters of pigs were treated with nonablative RF, and the histological response was analyzed in tissue samples. It was verified that even after three months of applying the technique, the function of the sphincter muscles was preserved and histological changes occurred in the muscle fibers with hyperplasia and hypertrophy due to the increase in type I collagen relative to type III [15]. Although the technique was not tested in humans in the perianal region, this mode of application was analyzed in the external urethral meatus [16], with a reduction in urine losses. Furthermore, the application of this technique to the female external genitalia improved the appearance and had a positive effect on sexual function [18].

Fecal incontinence is known to have an important effect on patients because of the inconvenient need to use protective or absorbent undergarments, which do not usually eliminate the odor and cause embarrassment. Elimination of the need for protection in four out of seven participants indicates a promising therapeutic response to the applied technique, which was confirmed by the effects on lifestyle, behavior, and depression parameters. We believe that the positive result of the fecal incontinence-related QoL in the current study was associated with a decrease in fecal losses.

Considering the above, the fecal incontinence-related QoL improvement after the RF sessions may have influenced the increased involvement of participants in daily activities such as reduced fear of going out, visiting relatives and friends, and using public transportation, and improved their psychological state. The psychological state and QoL of participants were likely mainly improved because they no longer feared the possibility of fecal incontinence incidences with the associated unpleasant or undesirable odors. We inferred that the sensation of improved fecal losses following treatment with the nonablative RF contributed to the willingness to confront the restrictive and negative thoughts and the perspective of feeling well.

The analysis of the ablative RF technique in a study with eight patients with AI revealed that seven women and one man, with a mean age of 59 years, showed improvements in the constraint parameters [25]. Another study with 16 patients (15 women and one man), with a mean age of 72.8 years, revealed that the depression parameter did not show improvement [26]. Studies conducted by Efron et al. [27] and Walega et al. [28], in which the majority of participants were 30 to 80 years old, showed immediate satisfactory results in all items of the FIQL.

Another effect highlighted in this pilot study was the satisfactory clinical response in the group of patients studied, based on the improvement of the AI severity. In a 2009 study, the improvement in severity was considered not statistically significant [28]. Data from another study using the ablative RF technique in eight participants showed no improvement in AI severity and no resolution of fecal losses. In addition, the study also demonstrated that ablative RF is used at a power of 465 kHz, 2-5 W, and a temperature of 85°C [25]. In this application of ablative transfer RF, needles are inserted in the anal region, which promotes thermal lesioning of the mucosa or anal sphincter. The lesions in the anal region may be associated with complications such as local hematoma, fever, and necrosis of the sphincter and anal mucosa [9,25-28].

The evaluation of the objective data and adverse effects indicated patient satisfaction. It should be noted that evidence-based medicine advocates the evaluation of satisfaction as a clinical response to the treatment performed. Based on the results of previous studies, AI loss was improved by ablative treatments, but it did not improve satisfaction. Our study demonstrated a high level of satisfaction since most participants who were treated with nonablative RF in the perianal region were satisfied. In other studies, using ablative transfer RF, this was not verified [9,25]. The associated side effects of treatment of AI with ablative RF, such as hemorrhage, anal pain, lesion, and anal bleeding, have led to the hypothesis that it does not show positive results in terms of satisfaction. Furthermore, in a study of eight participants (seven women and one man) aged 28-73 years, five reported dissatisfaction with ablative RF treatment because of complications such as pain and bleeding [25]. Another study with 10 women aged 44-74 (55.9 ± 9.2) years did not present any complications, but the authors identified through follow-up interviews that the satisfaction reported by the participants did not represent a significant score [9].

One patient in our study (participant 9) reported being dissatisfied, but it is important to emphasize that this patient presented with a depressive diagnosis and was on controlled medication. We believed that this pathology (depression) may have caused the negative response and unsatisfactory perception of this patient to the treatment response with nonablative RF. However, we did not question whether AI caused depression or vice versa, since it may have been developed because of incontinence. According to the Diagnostic and Statistical Manual of Mental Disorders V (DSM-V), depression is characterized as a disease that affects various aspects of a person’s life, including mood, thoughts, health, and behavior. The satisfaction corresponds to a pondering judgment between experiences, resulting from cognitive processes with the integration of affective elements [29].

The adverse effects observed after the nonablative technique indicated its safety as no analgesics or antibiotics were required, and other effects were considered minimal compared to the adverse effects of ablative RF. Ablative RF is a strategy used in the treatment of AI, but an intra-anal electrode with high temperatures is used, which causes microlesions in the anal sphincter. Furthermore, local anesthetics are required, and antibiotics are used during the recovery period. This method increases pain, bleeding, and discomfort during application [9,25-28].

Previous studies using the ablative RF technique to treat AI have reported different results from this present study [9,25-28]. The results of studies using ablative RF showed adverse effects such as bleeding, pain, diarrhea, and edema in the anal region and the use of medication by the participants [9,25-28]. The adverse effects reported in this study using nonablative RF treatment such as burning sensation, pruritus, and moist and sandy sensation in the anal region could be explained by the thermal effect, which facilitated the return of fluids outside the interstitial space and increased vascularization of the tissues and intradermal medium [14,30]. It is important to note that even with the reports of adverse effects in this study, the nonablative RF sessions did not need to be terminated. However, larger studies are needed to verify the adverse effects reported.

As a limitation of the study, the type of losses should have been assessed after the treatment. This is being corrected in the randomized controlled trial that is being conducted by the group.

## Conclusions

The results of the phase 1 clinical study appear promising, considering the reduction of fecal loss, participant satisfaction with treatment, and improved lifestyle, behavior, and depression symptoms. Also, the current research brings a non-invasive and low-cost treatment method, offering contributions. Finally, our study demonstrated minimal adverse effects.
